# Polyphosphate fertilizer impacts the enzymatic and non-enzymatic antioxidant capacity of wheat plants grown under salinity

**DOI:** 10.1038/s41598-023-38403-3

**Published:** 2023-07-11

**Authors:** Aicha Loudari, Salma Latique, Asmae Mayane, Gilles Colinet, Abdallah Oukarroum

**Affiliations:** 1 Plant Stress Physiology Laboratory, Mohammed VI Polytechnic University (UM6P)-AgroBioSciences, Lot-660 Hay Moulay, Rachid, 43150 Ben Guerir, Morocco; 2 Terra Research Center, Liege University-Gembloux Agro Bio Tech Faculty, 5030 Gembloux, Belgium; 3 High Throughput Multidisciplinary Research Laboratory, Mohammed VI Polytechnic University (UM6P), 43150 Ben Guerir, Morocco

**Keywords:** Plant sciences, Plant physiology, Plant stress responses

## Abstract

By 2050, the predicted global population is set to reach 9.6 billion highlighting the urgent need to increase crop productivity to meet the growing demand for food. This is becoming increasingly challenging when soils are saline and/or deficient in phosphorus (P). The synergic effect of P deficiency and salinity causes a series of secondary stresses including oxidative stress. Reactive Oxygen Species (ROS) production and oxidative damage in plants caused either by P limitation or by salt stress may restrict the overall plant performances leading to a decline in crop yield. However, the P application in adequate forms and doses could positively impact the growth of plants and enhances their tolerance to salinity. In our investigation, we evaluated the effect of different P fertilizers forms (Ortho-A, Ortho-B and Poly-B) and increasing P rates (0, 30 and 45 ppm) on the plant's antioxidant system and P uptake of durum wheat (Karim cultivar) grown under salinity (EC = 3.003 dS/m). Our results demonstrated that salinity caused a series of variations in the antioxidant capacity of wheat plants, at both, enzymatic and non-enzymatic levels. Remarkably, a strong correlation was observed between P uptake, biomass, various antioxidant system parameters and P rates and sources. Soluble P fertilizers considerably enhanced the total plant performances under salt stress compared with control plants grown under salinity and P deficiency (C+). Indeed, salt-stressed and fertilized plants exhibited a robust antioxidant system revealed by the increase in enzymatic activities of Catalase (CAT) and Ascorbate peroxidase (APX) and a significant accumulation of Proline, total polyphenols content (TPC) and soluble sugars (SS) as well as increased biomass, Chlorophyll content (CCI), leaf protein content and P uptake compared to unfertilized plants. Compared to OrthoP fertilizers at 45 ppm P, Poly-B fertilizer showed significant positive responses at 30 ppm P where the increase reached + 18.2% in protein content, + 156.8% in shoot biomass, + 93% in CCI, + 84% in shoot P content, + 51% in CAT activity, + 79% in APX activity, + 93% in TPC and + 40% in SS compared to C+. This implies that PolyP fertilizers might be an alternative for the suitable management of phosphorus fertilization under salinity.

## Introduction

The predicted global population is set to reach 9.6 billion by 2050, which highlights an urgent need to increase crop productivity to meet the growing demand for food and nutrition^1^. Nevertheless, this is becoming increasingly challenging due to changing climatic conditions^2^. Arid and semi-arid regions are characterized by high evapotranspiration levels that surpass precipitation, limiting water passage through the profile and, therefore, the soil’s capacity to leach salts^3^. This often causes soil salinity and results in low fertility of salt-affected soil^4^. Soil salinity is the accumulation of soluble salts on the soil surface or in subsurface layers^5^. Consequently, the increased levels of toxic ions such as sodium and chlorine (Na^+^ and Cl^−^) negatively impact plant growth and yield by limiting water uptake and causing ion toxicity, which can lead to nutrient imbalances^6^. This has a significant effect on the root’s capacity to absorb water and results in stomatal closure altering CO_2_ assimilation^7^ and therefore photosynthesis efficiency^8^. Later, an imbalance of the photochemical phase takes place in chloroplasts, causing a series of secondary stresses including oxidative stress^9,10^ where the continued exposure results in plant death^11^.

During oxidative stress, harmful products are generated in plants. Singlet oxygen (^1^O_2_), hydroxyl radicals (OH·) superoxide radicals and hydrogen peroxide (H_2_O_2_) are reactive oxygen species (ROS) produced when oxygen molecules are reduced^12^. In this regard, ROS are formed in plant cells as a result of electron leakage during respiration and photosynthesis^13^. Furthermore, these oxygen radicals are overproduced in salt-stressed plants due to changes in cellular water potential^14^, which affects cellular homeostasis^15^ and causes oxidative injury by the oxidation of DNA, proteins, lipids and other cellular components^16^. ROS has different sources and sites of production including chloroplasts, peroxisomes, mitochondria, the endoplasmic reticulum, and plasma membranes^17^. However, in photosynthetic organisms, the most important production location of ROS is the reaction centres of photosystems I (PSI) and II (PSII) in chloroplast thylakoids which are rich in reductants, oxygen, and high-energy intermediates^18^.

Although salinity tolerance mechanisms vary remarkably among plant species and even within different accessions of the same species^11^, salt-stressed plants adopted multiple physiological and metabolic strategies to minimise the damage caused by oxidative stress: Ion transport and absorption, ion compartmentalization, hormonal modulation, osmoprotectants and solutes biosynthesis, the synthesis of antioxidant compounds and the activation of antioxidant enzymes^5,19^. Furthermore, it is important to note that ROS are not known only as toxic metabolic products causing damage to cellular components, but also play a role in signal transduction to support the cell defence against the oxidative stress damage in cell compartments of stressed plants^20^ where the fast increase in their concentration is known as “oxidative burst”^18^. Additionally, their accumulation in plants under stress is a function of the ratio of ROS generation to ROS scavenging^18^. This balance can be disrupted by changes in growth circumstances, stress intensity, or stress duration^12,21^. Thus, boosting the plant’s antioxidant activity is necessary to minimize loss from ROS^20^. Indeed, plants have an efficient antioxidative system that includes both enzymes and non-enzymatic components to regulate ROS production and scavenging, therefore protecting cells from the overproduction of ROS and its consequences to guarantee good tissue and organ function^22^. In this regard, plants enhance the endogenous levels and the activity of the enzymatic ROS detoxification system which includes catalase (CAT), glutathione peroxidase (GPX), ascorbate peroxidase (APX), and superoxide dismutase (SOD) enzymes to neutralize ROS produced during oxidative stress^16,23^. These enzymes catalyse the conversion of superoxide anion (O_2_^−^) to hydrogen peroxide (H_2_O_2_)^14^ where SODs act as the initial line of defence against ROS^18^. In addition, plants also involve non-enzymatic compounds such as carbohydrates, proteins, ascorbic acid (AsA) phenolic compounds, flavonoids, and carotenoids to boost ROS detoxification^24^. These compounds effectively reduce cell oxidative damage by minimizing ROS activity or by working together with the enzymatic system to optimise the antioxidant performance using H_2_O_2_^16^.

Understanding the role of plant nutrition in the mitigation of salinity effects is one of the most important research areas in the actual context of climate change to meet the growing demand for food^25^. Durum wheat, a cereal crop of significant economic and agronomic importance, is extensively cultivated in the Mediterranean basin, with Morocco being the third-largest country in terms of wheat cultivation area, averaging about 24 million quintals per year from 2018 to 2021^26^ and about 45% of its rate of cultivation is in arid and semi-arid regions^27^. These regions have calcareous agricultural soil with a high deficiency in the available phosphorus, which is a challenging limitation that affects wheat productivity^28^. Phosphorus (P) is the second-most essential nutrient for plant growth and development after nitrogen (N), P plays an important role in plant metabolic functions, redox reactions, signal transduction, and carbohydrate metabolism^29^. Indeed, all essential processes such as photosynthesis, respiration, plant growth and development are severely affected by P deficiency^30^. Salinity has been shown to severely impact its bioavailability and mobility in the plant-soil system^31–33^. The rise in the electrical conductivity of saline soils not only alters their chemical and physical characteristics but also inhibits soil microbial biomass and alkaline phosphatase activity, which hampers P transformation in soil and its uptake by crops, reducing the chemical and biological accessibility of P. As a result, the P shortage in salt-stressed plants is exacerbated^34^. In this regard, it has been reported that plant growth was significantly inhibited under P deficiency due to the reduction in photosynthetic performance and nutrient uptake^32^, leading to ROS generation and oxidative stress^35^. Hence, any oxidative damage in plant cells caused either by P limitation or salt stress may restrict the overall plant performances which results in a decline in crop yield affecting food security around the world^36^.

Although the P use efficiency (PUE) differs depending on plant species, the severity of salinity stress in the rhizosphere and growing conditions^37^, most findings agreed that salt stress limits phosphorus accumulation in the tissues of salt-stressed plants^38,39^. However, salt-stressed plants showed positive results after the optimisation of P nutrition in salt-affected soil^31–33,38,40,41^. Additionally, it is important to state that phosphorus absorption by plants differs depending on the physicochemical parameters of the soil^8^, the exudation and architecture of roots^42^, the method and frequency of P fertilizer application^43,44^, and the microbial rhizospheric activity^45,46^. In this regard, Kohler et al.^47^ found that the salt tolerance of lettuce plants was enhanced by *Pseudomonas mendocina* resulting in a decrease of catalase activity with an enhancement in the dry weight of leaves and proline concentration. The same study indicates that *P. mendocina*’*s* ability to catabolize various substances, colonize roots effectively, and produce numerous enzymes and metabolites has a direct correlation with its beneficial impact on enhancing the availability of P and other nutrients under salt stress conditions^47^.

Besides, Ul Aibdin et al.^2^ revealed the positive effect of the combined application of zinc lysine and biochar on the antioxidant system and nutrient uptake of salt-stressed wheat. Furthermore, several investigations concluded that phosphorus application plays a pivotal role in the enhanced salt tolerance of Quinoa^33^, Sorghum^39^, Tomato^48^, Barley^49^, Common bean^40^ and Sugar beet^41^. The foliar application of P to wheat plants^50^ and common bean plants^43^ growing in salt stress conditions also exhibited this beneficial impact, revealed by the improvement in overall plant performances. Indeed, the positive interaction between salinity and phosphorus nutrition was confirmed in plant growth and yield. Thus, there is an immediate need to concentrate on the reasonable application of P sources that are more efficient to deal with the limited P availability in agricultural soils and improve crop productivity in salt-affected soil.

Polyphosphates (PolyP) are defined as a condensed form of P-fertilizers well-known for the slow and continuous release of available phosphorus to plants in the soil^31,32,44,51^. Furthermore, it has been found that polyphosphates are multifunctional fertilizers compared to orthophosphates, able to supply plants with sufficient amounts of available P while improving the availability of other crucial micronutrients like iron (Fe), zinc (Zn), and manganese (Mn)^52,53^. The condensed fraction of PolyP is slowly hydrolyzed during the crop's development, which provides a continuous source of available P to the plants^51^. Hence, these properties make PolyP an excellent sustainable source of P to meet plant requirements and minimize phosphorus losses from soils over time. However, the plant responses to polyphosphate (PolyP) fertilizer supply under salt stress conditions are not widely studied compared to Orthophosphate. In this work, we asked if soluble P-fertilizers enable wheat plants to mitigate the negative effect of salinity through the improvement of the enzymatic and non-enzymatic antioxidant systems and if there are any differences between plant’s responses to OrthoP and PolyP application. Hence, we hypothesize that using different forms of soluble P-fertilizers at various P rates could have a positive effect on the antioxidant system of wheat plants (durum wheat) and boost ROS detoxification under salt stress conditions, which is crucial for plant adaptation to salt stress conditions. Two Orthophosphates and one polyphosphate were applied at different P rates. Afterwards, wheat plant growth, mineral content, and enzymatic and non-enzymatic antioxidant capacities were evaluated.

## Materials and methods

### Experimental site, fertilization, plant material and experimental conditions

The experiment was carried out at the Experimental Farm of Mohammed VI Polytechnic University (UM6P), Ben Guerir, Morocco, in open field conditions. In Ben Guerir, the temperatures averaged out to 19 °C, with a range of 0 °C and 45 °C during the growth season. The daily light intensity was about PAR 280 µmol m^−2^ s^−1^. The total amount of precipitation was 99 mm during December, October, May, March, and January. Karim cultivar is one of Morocco’s most cultivated varieties of durum wheat (*Triticum durum*), well-known for its precocity, its adaptation to the bour and irrigated zones, tolerance to rust and *Septoria* and medium straw production. The seeds were collected from SONACOS (National seeds corporation commercialization, Morocco).

The soil used in the experiment was collected from an agricultural land: Rass El Ain-Morocco (31°43′55.1″N 7°36′49.9″W) chosen for its moderate deficiency in assimilable P (P_2_O_5_ = 30.33 ppm) (Table 1). Before the experiment, soil samples were taken from a 20 cm layer of the selected soil and analysed to refine the treatments. According to the standards, we have undertaken three replicates for every analysis. The chosen soil was found to have common properties with the majority of soils in the R’hamna region soils (Table 1).

**Table 1:** Physical and chemical properties of the chosen soil.

Soil parameters	Unit	Value	Method of analysis
Soil texture			
Clay	%	15	NFX 31-107
Slit		26	
Sand		58	
EC ext1/5	dS/m	1.587	NF ISO 11265
pH_water	–	7.893	NF ISO 10390
P_2_O_5_	ppm	30.33	NF ISO 11263
K_2_O		228.3	NFX 31-108
N-NO_3_	mg/kg	54.017	SKALAR
N-NH_4_		7.893	
MO	%	3.11	NF ISO 14235
C_org_		1.806	–
CaCo_3_ total		2490	NF EN ISO 10693
C.E.C	meq/100 g	12	NFX 31-130
Na_2_O	ppm	1546.66	NFX 31-108
MgO		624	
CaO		6472	
Cu		0.71	NFX 31-121
Mn		11.04	
Fe		6.26	

The soil was sieved at 8 mm after air-drying. A thin layer of gravel (1 cm) was initially placed inside each pot to ensure drainage. Three doses of phosphorus (P) (0, 30 and 45 ppm of P) were chosen for each soluble P fertilizer: Ortho-A, Poly-B and Ortho-B. Based on soil analysis, crop requirements, residues from previous crops and recent past of fertilization^54^, the COMIFER approach (French Committee for the Study and Development of Reasoned Fertilization) was used to add deficit nutrients and determine the chosen P doses. Accordingly, the dose of 30 ppm was ideal for the growth and development of wheat plants without salt stress. Hence, the selected P rates were tested in a preliminary laboratory trial using different salinity levels. The results showed encouraging plant responses. Indeed, using different forms of P fertilizers (Ortho-A, Poly-B and Ortho-B) aims to assess the eventual contribution of the form of P fertilizers to these responses.

The orthophosphate fertilizers (OrthoP) employed in the experiment are phosphoric acid-derived fertilizers with potassium (K) (Ortho-A) or nitrogen (N) (Ortho-B), each having 100% OrthoP with 52% and 62% P_2_O_5_, respectively. Poly-B is a short-chain linear polyphosphate fertilizer (PolyP) containing 47% P_2_O_5_ with 100% of PolyP as tripolyphosphates. Ammonium nitrate and potassium sulphate were used to equalize the amounts of N and K for all treatments. According to wheat nitrogen, the total quantity was divided and applied at one leaf stage, at tillering, and at the stem elongation stage, respectively. The adjustments were also made for controls. The negative control (C−) includes unfertilized plants that have not been subjected to salt stress, whereas the positive control (C+) corresponds to salt-stressed and unfertilized plants (salinity and P deficiency).

In polyethene pots measuring 24 cm in diameter and 35 cm in length and filled with 10 kg of dried soil, ten uniform size and healthy seeds were sown. After plant emergence, six seedlings were retained with the same size and appearance. After the seedling’s establishment, the salinity level was progressively increased by adding saline water (with definite EC) to reach moderate salinity conditions (EC = 3.003 dS/m). With ten repetitions per treatment, the experiment was performed in a totally randomized design. The plants were irrigated during the experiment with rap water when soil moisture content fell to 60% of its original value. The monitoring of Soil EC and moisture was ensured using the HH2 WET sensor (Delta-T devices). Starting from 6 weeks after sowing (WAS), the measurements were done every two WAS. The plant samples were collected at the heading stage (Z68–Z71 of Zadok’s scale) which corresponds to 12 WAS according to our experimental conditions.

### Chlorophyll content index

Chlorophyll Content Index (CCI) was determined using a non-destructive portable chlorophyll meter (CL-O1, Hansatech instruments). The fully mature and expanded functional leaves were kept for one minute in the dark and the CCI was measured from the middle part of the leaf. At least 12 independent leaves were used to estimate this parameter for each treatment. The CCI was measured at 6, 8, 10 and 12 WAS. Treatments exhibited significant differences at the last measurement chosen to present the results.

### Biomass

The plants were collected according to the recommended procedures^55^. Plants were washed, separated into shoots and roots, and dried at 75 °C until the dry weight (DW) stabilized.

### Nutrient analysis

Using Inductively Coupled Plasma Optical Emission Spectrometry (Agilent 5110 ICP-OES, USA), the concentrations of N, P, K, and Na were analysed on a dry-weight basis.

### Total proline content

The proline content was determined according to the method of Bates et al.^56^. 0.1 g of leaves were ground in 2 mL of 40% methanol. After centrifugation (5000 rpm for 10 min). 1 mL of the extract was added to 1 mL of glacial acetic acid and 6 M orthophosphoric acid (3:2 v/v) and 25 mg of ninhydrin. The tubes were then incubated in a water bath for 1 h at 100 °C. The tubes were cooled, and then 5 mL of toluene was added to each tube, the absorbance was determined at 528 nm. The standard range was prepared in the same way from different dilutions of the proline stock solution.

### Total malondialdehyde (MDA) content

Leaf lipid peroxidation was measured by thiobarbituric acid (TBA) as described by Savicka and Škute^57^. Lipid peroxides were extracted from a fresh weight of 0.21 g of leaves with 0.5 mL of trichloroacetic acid (TCA 0.1%). After centrifugation (15,000×*g* for 20 min), the chromogen was formed by mixing 1 mL of supernatant with 2.5 mL of 0.5% (w/v) TBA prepared in 20% TCA. The mixtures were incubated at 95 °C for 30 min and stopped by an ice bath. After centrifugation (15,000×*g* for 30 min), the absorbance of the chromogen formed (TBA-MDA complex) was determined at 532 nm and 600 nm using a spectrophotometer. The quantity of MDA was calculated in ε = 155.0 mM^−1^ cm^−1^.

### Total polyphenols content (TPC)

50 mg of leaves were ground in 1 mL of 80% methanol at 4 °C. The homogenate was centrifuged at 19,000*g* for 20 min. The supernatant was used for the analysis of phenol content. The content of phenolic compounds in the extract was estimated by the method of Taga et al.^58^. 100 µL of supernatant was mixed with 2 mL of 2% Na_2_CO_3_ and allowed to stand for 2 min. After incubation, 100 µL of 50% Folin Ciocalteu's Phenol Reagent was added, and then the reaction mixture was mixed thoroughly and allowed to stand for 30 min at room temperature in the dark. Absorbance was measured at 720 nm using a spectrophotometer. Gallic acid was used as a standard with a concentration range of 10 to 200 mg/L. The phenolic content was expressed in gallic acid equivalent (AGE) per mg of dry matter.

### Total soluble sugars content

The extraction of soluble sugars was made by grinding 50 mg of leaves in 4 mL of 80% cold ethanol. The supernatant was recovered after centrifugation at 5000 rpm for 10 min.

The dosage of soluble sugars was carried out according to the method of Dubois et al.^59^. Carbohydrates dehydrate into derivatives of furfural in a hot acid medium and combine with phenol giving a pink-salmon colour. In test tubes, 1 mL of a 5% phenol solution and 5 mL of concentrated sulfuric acid were added to 1 mL of the supernatant. After vertexing, the tubes were left to cool for 5 min and then the optical density was measured at 485 nm. The soluble sugar contents were determined by reference to a standard range established by glucose solutions of known concentrations.

### Total soluble protein content

The total soluble protein content of the enzyme extracts was determined following the method of Bradford^60^, using Bovine Serum Albumin (BSA) as a protein standard.

### Preparation of plant extracts for enzyme activity assays

Enzyme extract was prepared for the assay of antioxidant enzymes by the method of Tejera et al.^61^. The extract was prepared by grinding 100 mg of fresh material with 2 mL of potassium phosphate buffer (0.1 M, pH 6) and 5% of insoluble polyvinylpyrrolidone, centrifuged at 12,000×*g* for 30 min and the supernatant was used for estimation of enzyme activity. The enzyme protein was determined by the method of Bradford^60^ for all the enzymes expressing the specific activity of enzymes.

### Enzyme activity assays

The activity of SOD (EC 1.15.1.1) was assayed according to the method described by Beyer and Fridovich^62^. The reaction mixture contained some modifications, by measuring the inhibition of the photochemical reduction of tetrazolium nitroblue. The reaction mixture contains 2550 µL of 100 mM phosphate buffer (pH 7.8), 75 µL of 55 mM methionine, 300 µL of 0.75 mM tetrazolium nitroblue, 60 µL of 0.1 mM riboflavin and 50 µL of the enzymatic extract. Riboflavin is added last, and the reaction was initiated by placing the tubes under 75 W fluorescent lamps. Illumination was started to initiate the reaction at 30 °C for 1 h. Identical solutions that were kept under the dark served as blanks. The SOD activity was measured at 560 nm. One unit of SOD activity was defined as the quantity of SOD required to obtain a 50% inhibition of the reduction of NBT. The activity was expressed as units per mg of protein content.

Catalase activity CAT (EC 1.11.1.6) was measured according to Chandlee and Scandalios^63^ and modified by Jaleel et al.^64^. The assay mixture contained 2.6 mL of 50 mM potassium phosphate buffer (pH 7.0), 0.4 mL of 15 mM H_2_O_2_, and 0.04 mL of enzyme extract. The decomposition of H_2_O_2_ was followed by the decline in absorbance at 240 nm. The enzyme activity was expressed in units per milligram protein (U = 1 mM of H_2_O_2_ reduction min^−1^ mg^−1^ protein).

Ascorbate peroxidase (APX) activity (EC 1.11.1.11) was determined according to Oidaira et al.^65^. The reaction mixture (1 mL) contained 50 mM potassium phosphate buffer (pH 7.0), 0.5 mM ascorbic acid, 0.1 mM H_2_O_2_, and 200 μL of enzyme extract. The absorbance was read as decreased at 290 nm against the blank. The enzyme activity was expressed in units per milligram protein (U = change in 0.1 absorbance min^−1^ mg^−1^ protein).

### Statistical analysis

Analysis of variance (ANOVA, one-way) and SPSS data processing software were used to conduct statistical analysis considering three independent replicates per treatment. Based on the ANOVA results, and for a 95% confutation level, a Duncan test was performed to compare the means. Significant differences between treatments were reflected by different letters in the figures. To determine the relationship between the various treatment groups and the biochemical characteristics of wheat plants, principal component analysis (PCA) was also carried out using SPSS and based on the pattern matrix with rotation method: Oblimin with Kaiser normalization.

### Collection of plant material

The plants were collected according to plant materials collection guide of Hoolehua Plant Materials Center^11^, USDA Natural Resources Conservation Service (NRCS).

## Results

### Biomass

**Figure 1:**
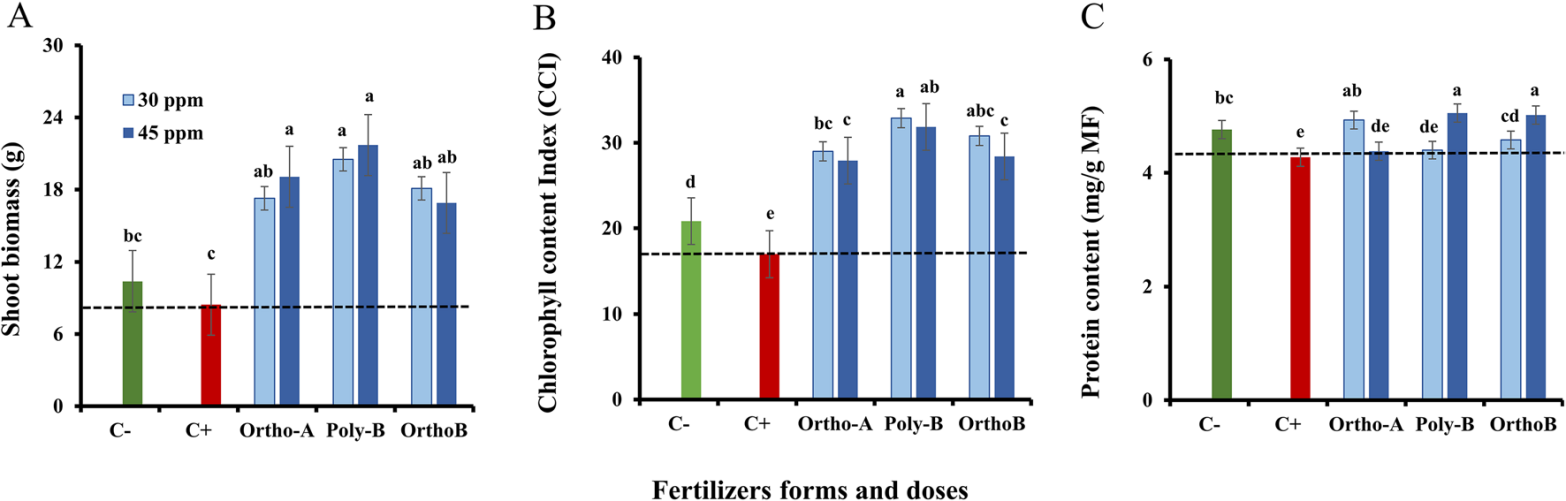
The effect of different soluble P-fertilizer forms (Ortho-A, Poly-B and Ortho-B) and doses (0, 30 and 45 ppm of P) on Shoot Biomass (**A**), Chlorophyll content index (CCI) (**B**) and Protein content (**C**) of wheat plants grown under salinity, measured 12 Weeks After Sowing (WAS). C+ salt-stressed and unfertilized plants, C−: unfertilized plants without salt stress. Statistical analysis was performed using one-way ANOVA and SPSS data processing software. Duncan’s test was used for the comparison of means. Treatments having a different letter(s) are significantly different at the 5% level.

Compared to unfertilized plants (C− and C+), the shoot dry weight (DW) significantly increased in fertilized plants (Fig. 1A). Plant responses were strongly influenced by the dose or the form of fertilizers. Indeed, shoot DW increased significantly at 30 ppm of P of Ortho-B (+ 114.2%) followed by Ortho-A (+ 125.6%) at 45 ppm of P in comparison with salt-stressed and unfertilized plants (C+). The increase in shoot DW reached + 156.8% using Poly-B fertilizer which showed the best performances for both P doses.

### Chlorophyll content index

After 12 weeks of growth, Chlorophyll Content Index (CCI) measurements showed significant differences between treatments. Unfertilized and salt-stressed plants (C+) revealed a decrease of − 22.6% in CCI in comparison with unfertilized and non-salt-stressed plants (C−) (Fig. 1B). Nevertheless, compared to control plants (C+ and C−), fertilized plants had a higher CCI. At 30 ppm of P, the enhancement in CCI parameter reached + 71%, + 81% and + 93% in plants fertilized with Ortho-A, Ortho-B and Poly-B, respectively compared to C+ (Fig. 1B). CCI was unaffected by P doses of the different P fertilizers. However, their form had a significant impact on this parameter at both 30 ppm and 45 ppm doses, where Poly-B showed a significant increase estimated by + 17.4% in comparison with Ortho-B and Ortho-A.

### Protein content

The significant effect of salt stress on protein content in leaves was revealed in unfertilized plants by a decrease of − 10.2% in C+ plants compared to C− plants. Poly-B and Ortho-B showed the best performance at 45 ppm of P with an increase of + 18.2% and + 17.4% in the plant protein content, respectively, while Ortho-A fertilizer indicated a positive response at 30 ppm of P where the protein content in plants increased by + 15.3% compared to C+ plants. The form and the dose of P-fertilizers strongly influenced this parameter (Fig. 1C).

### Shoot mineral content

**Figure 2:**
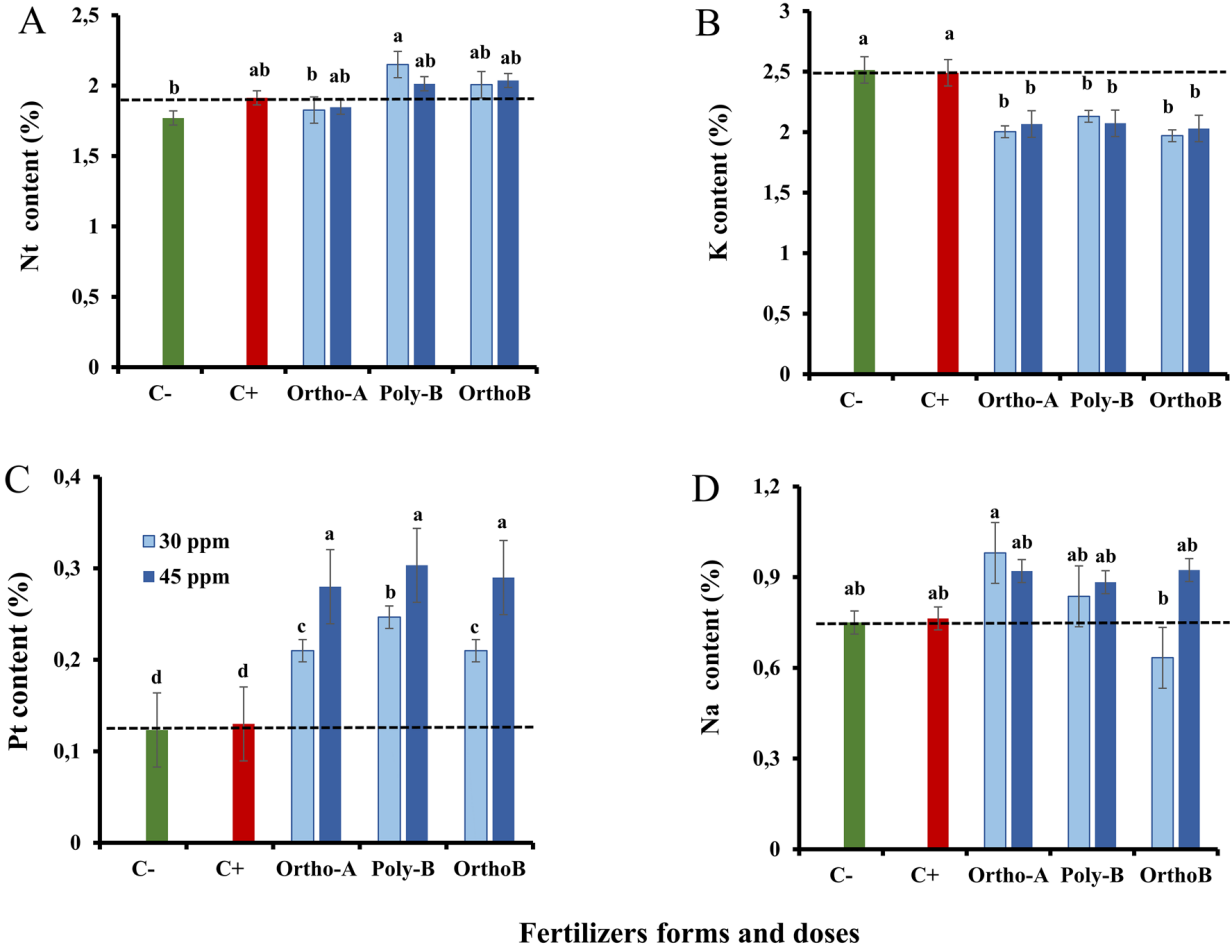
The effect of different soluble P-fertilizer forms (Ortho-A, Poly-B and Ortho-B) and doses (0, 30 and 45 ppm of P) on shoot mineral contents: Total nitrogen (Nt) (**A**), Potassium (K) (**B**), Total phosphorus (Pt) (**C**) and sodium (Na) (**D**) of wheat plants grown under salinity, measured 12 Weeks After Sowing (WAS). C+ salt-stressed and unfertilized plants, C−: unfertilized plants without salt stress. Statistical analysis was performed using one-way ANOVA and SPSS data processing software. Duncan’s test was used for the comparison of means. Treatments having a different letter(s) are significantly different at the 5% level.

Figure 2 shows the mineral content in shoots. The total amount of N in shoots (Nt) did not exhibit any difference between the C+ plants and the other P-treatments except for poly-B fertilizer at 30 ppm of P where a significant increase in Nt was estimated by + 21.5% and + 12.4% in comparison with C− and C+ plants (Fig. 2A). However, at 30 ppm of P, the Nt content significantly declined by − 17% in Ortho-A compared to Poly-B plants at the same dose, respectively. This response was, therefore form dependent (Fig. 2A).

However, compared to unfertilized plants, the potassium (K) content in shoots significantly declined by − 20% in fertilized plants, where similar responses were revealed for both doses (Fig. 2B). Here, the response was not affected by the dose or the form of P fertilizers. Furthermore, there are no significant differences in the K accumulation between control plants (C+ and C−) (Fig. 2B).

However, the total P content (Pt) in shoots significantly increased in fertilized plants compared to control plants (C+ or C−). The dose and the form of P fertilizers have a significant impact on the P accumulation of plants. The fertilized plants showed similar results at 45 ppm of P with significant enhancement in Pt content in comparison with the 30 ppm dose (Fig. 2C). Accordingly, the OrthoP fertilizers did not show any differences for both doses, where the P accumulation in shoots increased by + 115% and + 62% at 45 and 30 ppm of P, respectively compared to C+ and C− plants. The same tendency was detected using Poly-B fertilizer which revealed the highest significant effect on the total shoot P content estimated by + 131% and + 84% at 45 and 30 ppm of P, respectively compared to control plants (Fig. 2C).

The sodium (Na) content in shoots was improved in fertilized plants in comparison with salt-stressed and unfertilized plants (C+) which was unexpected. The increase was more relevant at 30 ppm of P using Ortho-A where Na content in shoots increased by + 28% compared to control plants (C+ and C) (Fig. 2D). Ortho-B fertilizer showed the lowest significant value of the Na accumulation in shoots, estimated by − 22% and − 56% compared to C+ and Ortho-A plants at 30 ppm of P. The effect was dose/form dependent.

### Antioxidant system

**Figure 3:**
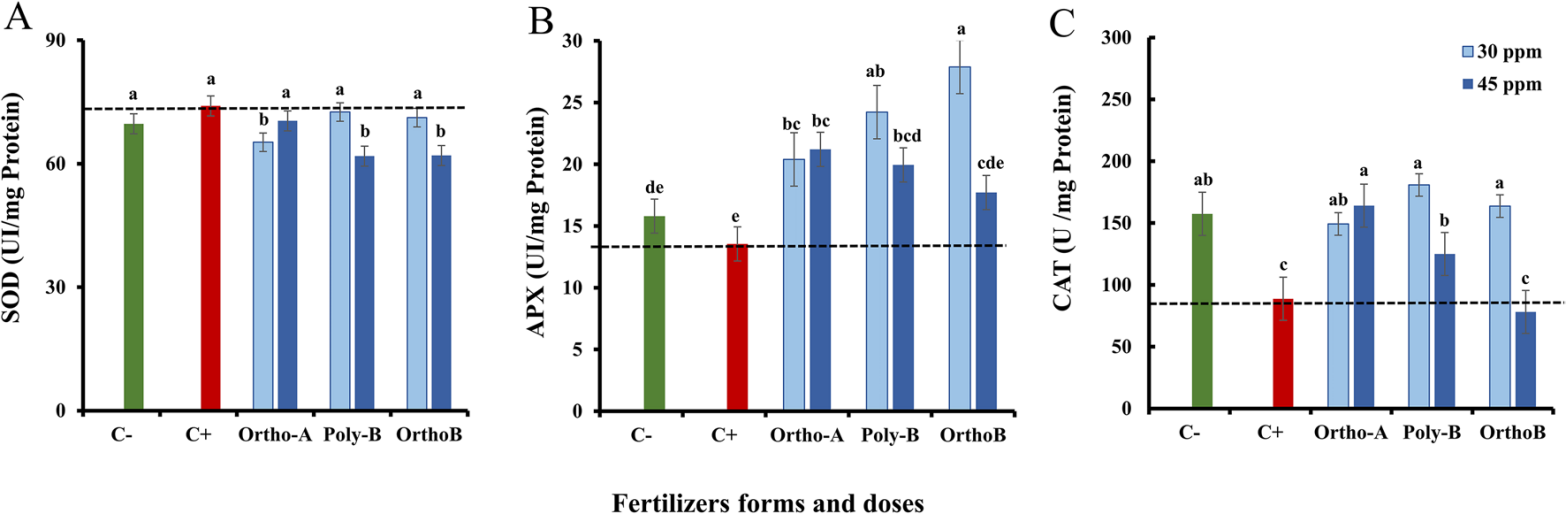
The effect of different soluble P-fertilizer forms (Ortho-A, Poly-B and Ortho-B) and doses (0, 30 and 45 ppm of P) on superoxide dismutase (SOD) (**A**), ascorbate peroxidase (APX) (**B**) and Catalase (CAT) (**C**) of wheat plants grown under salinity, measured 12 Weeks After Sowing (WAS). C+ salt-stressed and unfertilized plants, C−: unfertilized plants without salt stress. Statistical analysis was performed using one-way ANOVA and SPSS data processing software. Duncan’s test was used for the comparison of means. Treatments having a different letter(s) are significantly different at the 5% level.

The combined effect of two Orthophosphates (Ortho-A and Ortho-B) and one polyphosphate (Poly-B) at different P levels (0, 30 and 45 ppm) and the salt stress was also evaluated on the antioxidant system through the determination of enzyme activities (Fig. 3).

In the present study, the data in Fig. 3. show significant relationships between P-fertilizers and APX, CAT and SOD activities in all treatments.

Figure 3A shows the SOD enzymatic activity in leaves. The source of fertilizers has a significant effect on SOD activity, but it depends on the dose of P. Furthermore, the SOD activity in C− did not show a significant difference with the C+ plants. Accordingly, Ortho-A at 45 ppm of P, Poly-B and Ortho-B at 30 ppm of P had a similar response in the activity of SOD compared to C+ plants. Additionally, this response did not show a significant difference between each other compared to C+ plants. However, when the stressed plants were treated with Ortho-A at 30 ppm of P, Poly-B and Ortho-B at 45 ppm of P, the SOD enzymatic activity decreased significantly by − 11.9%, − 16.46% and − 16.26%, respectively, compared to C+ plants.

On the other hand, the significant effect of salt stress on the activity of APX in leaves was revealed in unfertilized plants by a decrease of − 14.2% in C+ plants compared to C− plants. Poly-B and Ortho-B showed the best performance at 30 ppm of P with an increase of + 79% and + 106% in the plant APX activity, respectively. Additionally, Ortho-A fertilizer indicated a positive response at 45 ppm of P where the activity of APX in plants increased by + 56.5% compared to C+ plants. The form and the dose of P-fertilizers strongly influenced this parameter as shown in Fig. 3B. However, the CAT activity in salt-stressed and unfertilized plants (C+) was found to be reduced by − 43.6% compared to the CAT activity measured in unfertilized plants without salinity stress (C−) (Fig. 3C). Additionally, fertilized plants showed an increase in CAT activity compared to C+ plants except for Ortho-B at 45 ppm of P which showed a significant decrease of − 13.6% compared to C+ plants. Furthermore, after 12 weeks of growth with a dose of 30 ppm of P, the CAT activity increased by + 51%, + 46%, and + 40% in plants fertilized with Poly-B, Ortho-B and Ortho-A, respectively compared to C+ (Fig. 3C). Notably, the difference between fertilizer forms was significant mainly for Poly-B which increased the CAT activity by + 51% at 30 ppm of P compared to C+ plants. This outcome demonstrated that the use of different fertilizer forms enhances the ascorbate peroxidase and catalase activities, and functions as a defence against ROS generated during salt stress.

**Figure 4:**
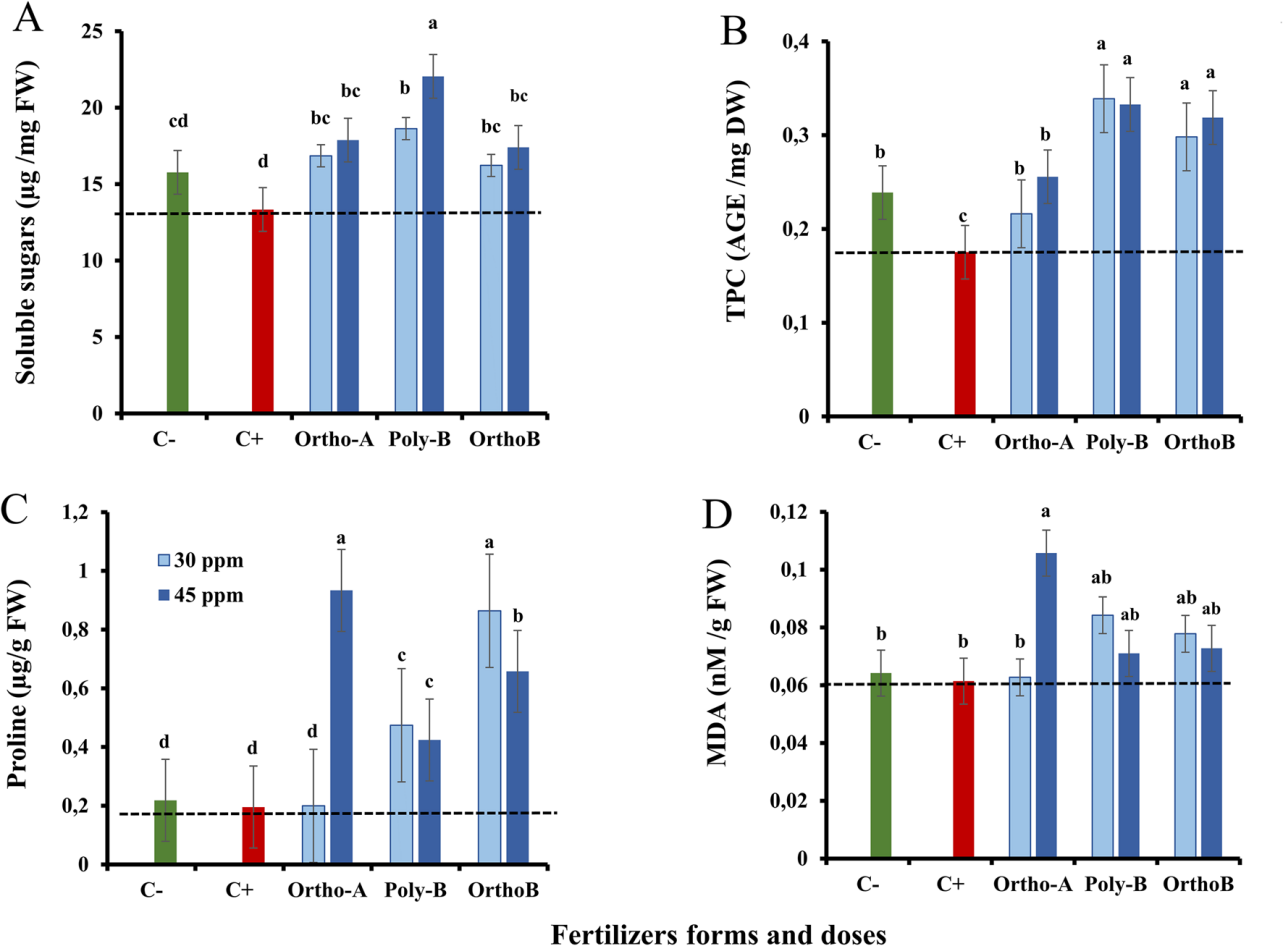
The effect of different soluble P-fertilizer forms (Ortho-A, Poly-B and Ortho-B) and doses (0, 30 and 45 ppm of P) on leaf content of Soluble Sugars (SS) (**A**), Total Polyphenols Content (TPC) (**B**), Proline (**C**) and Malondialdehyde (MDA) (**D**) of wheat plants grown under salinity, measured 12 Weeks After Sowing (WAS). C+ salt-stressed and unfertilized plants, C−: unfertilized plants without salt stress. Statistical analysis was performed using one-way ANOVA and SPSS data processing software. Duncan’s test was used for the comparison of means. Treatments having a different letter(s) are significantly different at the 5% level.

Overall, plants exhibit ROS defence mechanisms that incorporate the production of antioxidant molecules and enzymes^25^. Non-enzymatic antioxidants such as proline and polyphenols are known to be associated with the reduction of ROS, which assists to provide resistance to oxidative stress^66^. For these reasons, we investigated the combined effect of P-fertilizer forms (Ortho-A, Poly-B and Ortho-B) and doses (0, 30 and 45 ppm) on total soluble sugars (SS), total polyphenols content (TPC), proline and malondialdehyde (MDA) content (Fig. 4).

Therefore, the soluble sugars content (SS) in plant leaves of *Triticum durum* variety Karim was elevated proportionally with the increase of P fertilizer doses. The highest values of SS were recorded with Poly-B at 45 ppm of P (20.04 ± 1.931 µg mg^−1^ FW) compared to salt-stressed and unfertilized plants (C+) (13.34 ± 1.301 µg mg^−1^ FW). The lowest value of SS content was recorded with Ortho-B at 30 ppm of P (16.22 ± 2.806 µg mg^−1^ FW) compared to C+ plants.

Indeed, the results in (Fig. 4A) showed that wheat salt-stressed plants treated with different fertilizer forms and doses had increased soluble sugar content for all conditions compared to C+ plants.

Total polyphenols content (TPC) decreased by − 26.61% in salt-stressed and unfertilized plants (C+) compared to unfertilized plants without salinity stress (C−) (Fig. 4B). However, fertilized plants showed a significant increase in TPC compared to C− and C+ plants. Indeed, compared to C+, Poly-B and Ortho-B showed similar results in TPC with an increase of + 89% and + 82% at 45 ppm of P, respectively. The lowest significant value of TPC was obtained with Ortho-A at 30 and 45 ppm of P, with an increase of + 23% and + 46% respectively in comparison with C+ plants. Nevertheless, the highest significant value of TPC was obtained with Poly-B fertilizer at 30 ppm of P with an increase of + 93% compared to C+ plants.

The proline content in leaf extracts of wheat plants subjected to different fertilizer forms and doses is shown in (Fig. 4C). The proline value of Ortho-A at 30 ppm of P was the lowest (0.20 ± 0.024 µg g^−1^ FW) and not significant compared to the unfertilized plants without salt stress (C−). However, the application of Poly-B, Ortho-B at 30 and 45 ppm of P and Ortho-A at 45 ppm of P improved notably the proline content with an increase of + 142%, + 342%, + 117%, + 236% and + 377% compared to C+ plants respectively and the maximum was attained with Ortho-A at 45 ppm of P with an increase of + 377% compared to C+ plants.

Furthermore, in Fig. 4D, there was a non-significant increase in the malondialdehyde (MDA) accumulation observed in fertilized wheat plants compared to both salinity-exposed (C+) and non-exposed (C−) unfertilized plants, except for Ortho-A. However, a significant increase in MDA accumulation was observed specifically in plants treated with Ortho-A fertilizer (+ 72%) at 45 ppm of P in comparison with C+ plants.

### Principal component analysis (PCA)

**Figure 5:**
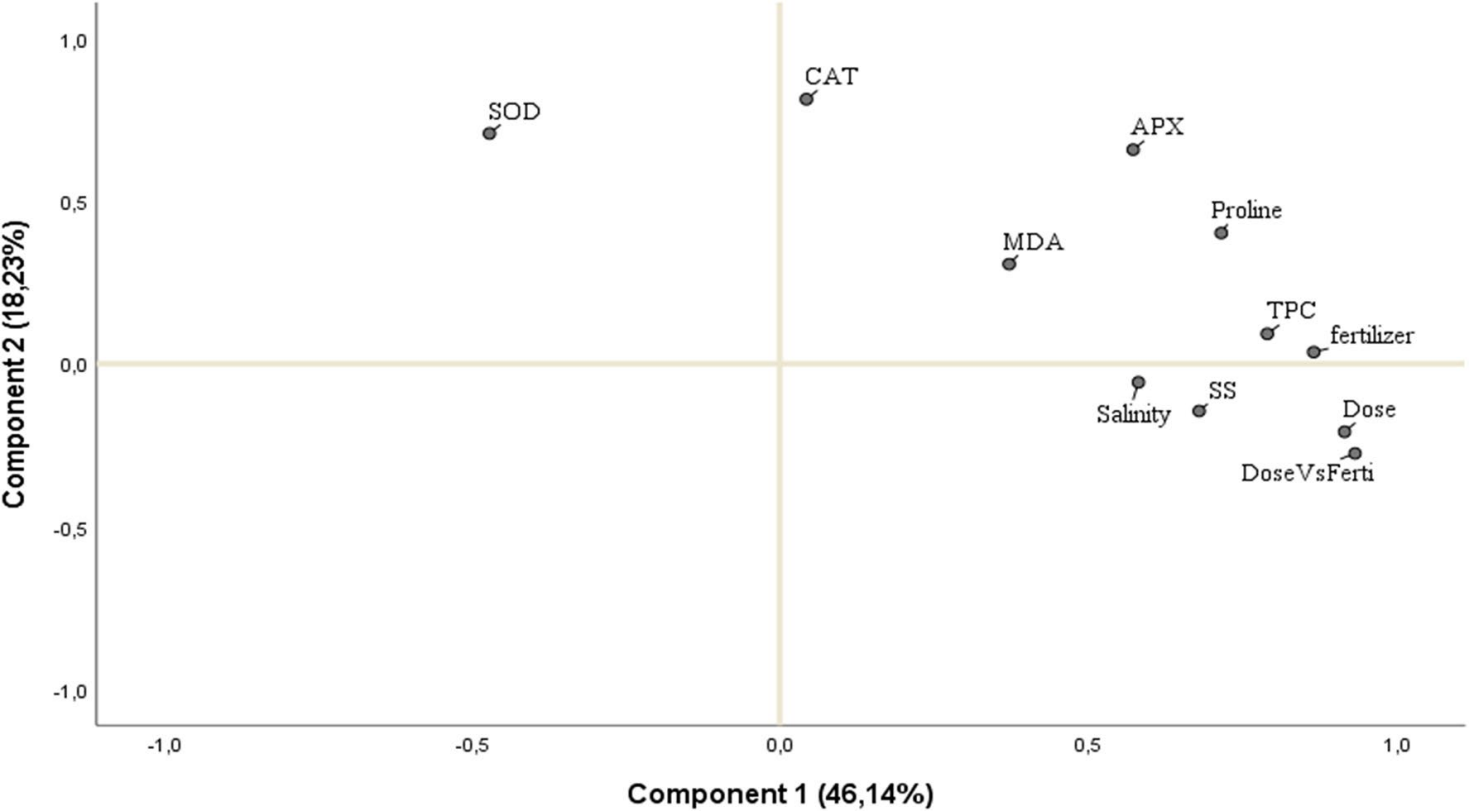
Principal component analysis (PCA) for the evaluation of the interactions between the various treatment groups and the biochemical parameters of the wheat plants grown under salinity using IBM SPSS and based on the pattern matrix with rotation method: Oblimin with Kaiser normalization. *CAT* catalase, *APX* ascorbate peroxidase, *SOD* superoxide dismutase, *TPC* Total Polyphenols Content, *MDA *Malondialdehyde, *SS* Soluble Sugars. Combined effect of fertilizer forms and doses (Dose vs Ferti).

To evaluate the interactions between the various treatment groups and the biochemical parameters of the wheat plants, principal component analysis (PCA) has been used (Fig. 5). Components 1 and 2 of the PCA in Fig. 5 together explained 64.37% of the overall variation.

Based on the pattern matrix with rotation method: Oblimin with Kaiser normalization, it was observed that salinity, fertilizer doses and forms, total soluble sugars (SS), total polyphenols content (TPC), proline, the combined effect of fertilizer forms and doses, (Dose Vs Ferti) and malondialdehyde (MDA) were correlated to component 1, however, SOD, APX, CAT were correlated to component 2. Globally, our results confirm that the salinity stress has a negative effect on soluble sugar accumulation but a positive effect on the activation of the following enzymes, for example, CAT and APX.

Nevertheless, the fertilizer treatment shows a significant effect on plants grown under salt stress by enhancing TPC, proline, MDA and especially the activity of the following enzymes: catalase (CAT) and ascorbate peroxidase (APX). This means that the fertilizer form could be used as a promising plant growth biostimulant for treating wheat plants (variety Karim) cultivated under salt stress.

## Discussion

It is well known that ROS formation is one of the most detrimental effects of salinity on plants which can disrupt several physiological functions and harm biological components^16^. In our investigation, parameters related to the plant's antioxidant system and P uptake are highlighted to reveal the complex relationship between salinity and P in wheat plants. Accordingly, it has been reported that plant growth reductions and salt toxicity symptoms were less severe when P nutrition was adequate and were more pronounced by P shortage^40^. Indeed, all measured parameters indicate that the salt tolerance and plant growth were improved after the application of the P-soluble fertilizers. Furthermore, this beneficial effect has been shown in different plant species grown under salinity and supplied by contrasting P doses^31–33,39,40,48,67,68^.

The salinity effects on the photosynthesis process can range from the restriction of the CO_2_ diffusion to chloroplasts via stomatal opening limitation up to substantial changes in the carbon metabolism and leaf photochemistry, or they can induce oxidative stress by stimulating the production of reactive oxygen species (ROS)^24^. Previous studies have reported that oxidative stress appears in plants exposed to salinity as a secondary effect and can cause significant changes to the photosynthetic machinery^36,69^. In the present work, the decrease in chlorophyll content index (CCI) observed in salt-stressed plants reached − 23% in unfertilized and salt-stressed plants (C+) compared to unfertilized plants grown without salinity (C−) (Fig. 1B). This was also described in several investigations focused on the impact of salt stress on plants^36,70^. In this regard, the essential functional chloroplast protein complexes (photosystem I (PSI) and II (PSII), ATP-synthase, and Cytb6f) involved in collecting light energy are seriously impacted in plants grown under salinity, as revealed by Dekker and Boekema^71^. Hence, this decrease might be linked to both the instability of pigment-protein complexes which limit the biosynthesis of chlorophyll^30^ and the rise in the activity of chlorophyllase, an enzyme degrading the chlorophyll^72^. Besides, the reduction of chlorophyll content observed in salt-stressed plants may also be caused by the generation of ROS^16,36^ since the electron transport chain (ETC) in the chloroplasts is the principal generator of ROS^18^. This provokes serious structural changes in the light-harvesting complex^73^, alters the light fixation capacity and hence lowers the photosynthetic efficiency of plants^35^. Thus, the regulation of the ROS state in the chloroplast is essential for the survival of plants under stressful conditions^12^. However, the level of ROS in plants varies depending on the environmental and physiological conditions the plant is experiencing at the time^20,24^. As a result of prolonged stress, photosynthetic metabolism is downregulated, which causes changes in leaf biochemistry in response to a decrease in net CO_2_ assimilation^69^. In such circumstances, plants would often employ defensive mechanisms, such as increasing their water use efficiency (WUE) and controlling the rate of transpiration and the net CO_2_ levels in their leaves^36^. Accordingly, stomatal closure has a direct impact on plant growth and development under salt stress conditions, by preserving water in the plant tissue, reducing water loss through transpiration, and inhibiting cell expansion which ultimately leads to a reduction in plant biomass^68,74^. In addition, long-term exposure to salinity or/and P shortage interferes with metabolic mechanisms that regulate gas exchange, such as the triose phosphates regeneration, Rubisco and Ribulose-1,5-bisphosphate (RuBP) activities^35^. Furthermore, a recent study by El-Mejjaouy et al.^75^ reported that phosphorus shortage severely disrupts CO_2_ assimilation. Thus, previous investigations have supported the need for appropriate phosphate nutrition in salt-stressed wheat for efficient ion compartmentation, namely through the contribution to the efficient partitioning of C and the photo-assimilates use^50,76^. In line with this, Mohamed et al.^40^ concluded that the increased CCI in fertilized plants improves photosynthetic activity, which leads to optimal plant growth and higher biomass production. Our results agree with this conclusion since we found that the CCI rose by + 71%, + 81% and + 93% in fertilized plants treated with Ortho-A, Ortho-B and Poly-B, respectively, compared to salt-stressed and unfertilized plants (C+) (Fig. 1B). CCI was not significantly affected by the P doses, but there was a substantial difference across fertilizer types, especially for Poly-B at 30 ppm P, which rose by + 17.5% compared to Ortho-B and Ortho-A at 45 ppm P (Fig. 1B).

According to research published by Fahad et al.^77^, plants grown under moderate salt stress conditions spent more energy on defence mechanisms than on the production of biomass. In our study, shoot biomass was reduced in response to salt stress (Fig. 1A) which is in line with previous reports in the literature^32,33,36,78^. Correspondingly, it was reported that shoot growth could be a more relevant parameter for evaluating the tolerance of salt-stressed plants than root growth. We presume that the decline in dry biomass was responsible for the reduction observed in chlorophyll content and stomatal closure^32,74^. Furthermore, it has been proven that biomass production and photosynthetic capacity are positively correlated for maize plants^9^, wheat^32^, pepper^79^, and quinoa^10^ grown under salinity. Hence, salt-stressed plants evolved phenotypic flexibility to minimize the detrimental effects of salinity^80^. In the present work, the decline in biomass may be a plant survival mechanism in response to a failure in carbon (C) assimilation^72^. Several studies have demonstrated the pivotal role of phosphorus nutrition in plant growth, and how reduced phosphorus absorption under salt stress may inhibit shoot and root biomass^33,38^. This agreed with our findings which showed a significant decline in shoot biomass of salt-stressed and unfertilized plants (C+) compared to salt-stressed and fertilized plants (Fig. 1A). Additionally, our results demonstrated that not only the form of P-fertilizers has a substantial impact on this parameter but also P doses. Shoot biomass was significantly enhanced by + 157% using Poly-B fertilizer at both doses compared to C+, followed by Ortho-A (+ 126%) at 45 ppm P and Ortho-B (+ 114%) at 30 ppm P (Fig. 1A). Accordingly, it was noted that a sufficient supply of P boosted the vegetative growth and the development of robust root systems, which are essential for the effective nutrient uptake from the soil^70^. Nevertheless, a high concentration of P-soluble fertilizers in the soil (60 ppm) had a negative impact on wheat under salinity (data not shown). The same tendency was also found in other crops such as soybean^81^, barley^49^ common bean^67^, and maize^82^. Poly-B fertilizer exhibited the best performances in this parameter at both doses (Fig. 1A). This could be associated with the increased availability of phosphorus in the soil solution, which may be the result of the gradual and continuous release quality that polyphosphate possesses^52^. In line with this, a recent study found that polyphosphate fertilization significantly improved the P uptake of maize plants^53^ where the total dry biomass and P uptake were shown to be significantly correlated (r = 0.91) in the presence of polyphosphate^53^. The same research indicated that the progressive release of available P in the soil using PolyP may result in an improvement of P uptake of plants which supports previous studies by Behdad et al.^78^, Muhammad et al.^36^, Bouras et al.^41^, Loudari et al.^32^. In this regard, it was shown that P-application aided in the development of a robust root system in lentil plants, maximizing their capacity to absorb other nutrients (e.g.: nitrogen, calcium, and potassium)^83^. Thus, their content in plants was significantly enhanced after P fertilization^48,52,83^. Our findings agree with this statement since we found that the total nitrogen (Nt) increased in shoots of fertilized plants grown under salt stress compared to control plants. The increase reached + 21.5% and + 12.4% of Nt content in comparison with C− and C+ plants, respectively (Fig. 2A). The plant response was form dependent since the Nt content significantly declined at 30 ppm P by − 17% in Ortho-A compared to Poly-B plants at the same dose, respectively (Fig. 2A).

The present study showed also that the protein content in leaves decreased under salt stress mainly for C+ plants (Fig. 1C), which implies that salt stress and P deficiency have a cumulative effect over time. The observed decline suggests that under our growth conditions (salinity and P shortage), the plant vitality was somewhat reduced. Indeed, our findings indicated that fertilized plants grown under salinity showed the best performances in protein content increased by + 18.2% and + 17.4% for Poly-B and Ortho-B at 45 ppm P, respectively, followed by Ortho-A at 30 ppm P where this parameter increased by + 15.3% compared to C+ plants (Fig. 1C). Here, the plant’s response was form/ dose dependent.

In our investigation, no significant difference between P treatments in the total amount of P (Pt) in shoots, especially at 45 ppm P. However, OrthoP fertilizers (Ortho-A and Ortho-B) exhibited similar responses for both doses (+ 115% and + 62% at 45 and 30 ppm P, respectively) (Fig. 2C). Thus, the plant response was dose dependent. Compared to C+, salt-stressed plants fertilized with Poly-B showed high values in shoot Pt content (+ 131% and + 84% at 45 and 30 ppm P, respectively) (Fig. 2C). Correspondingly, the findings of Gao et al.^53^, have also recently verified this statement. Interestingly, the authors concluded that the application of polyphosphates considerably improved the absorption of phosphorus (60 kg P ha^−1^) in the roots and shoots of maize plants^53^. Therefore, the use of polyphosphate fertilizers, which increases progressively the amount of available phosphorus in the soil could be the reason for the enhanced P uptake of plants^31,32,42,52,53^. According to recent reports, the enhanced phosphorus absorption under Poly-B application might be linked to its slow and progressive hydrolysis caused by the release of enzymes that hydrolase P and the acidification of the rhizosphere, supposing that both processes are involved in polyphosphates hydrolysis^51,52^. Furthermore, the observed increase in Pt content in wheat plants under salinity and P-fertilization (Fig. 2B) might be the result of a synergistic action of sodium, involved in the acquisition of P and/or its transport to shoots^84^. Nevertheless, Phang et al.^81^ found that high concentrations of phosphorus in the medium promoted Na uptake and decreased the soybean's tolerance to salt stress which was in line with our findings where the Na accumulation in fertilized plants grown under salinity was considerably important than in salt-stressed plants without any P supply (C+) (Fig. 2D). In this regard, it has been demonstrated that the reduction in plant growth and development under salt stress conditions might be due to a nutritional imbalance as well as an excessive acquisition of sodium^85^. Interestingly, the green alga *Chara corallina*'s ability to acquire phosphorus is shown to be highly dependent on the availability of sodium in the medium^86^. The same study showed that P/Na co-transport is supported in this species since the P influx is stimulated by Na, and the Na influx is stimulated by P. Accordingly, the P acquisition was improved in *Zostera marina* L. by Na, as reported by Rubio et al.^87^, which suggests the contribution of a Na-dependent high-affinity phosphate transporter in the roots and shoots of this plant. Furthermore, Talbi et al.^49^ observed that salt stress considerably raised the P content in shoots of *Hordeum maritimum* plants when associated with a high P application, but there is no significant impact on this parameter when salinity was combined with P shortage. This statement was confirmed in our study by the increased P content in salt-stressed and fertilized plants compared to salt-stressed and unfertilized ones (C+) (Fig. 2C). Accordingly, in a recent study, a significant rise in P and K use efficiencies was observed in *Aeluropus littoralis* plants grown under salt stress and P deficiency conditions, which suggests that there is an efficient transfer of phosphorus from older organs to more youthful tissues in full growth activity^68^.

Potassium's roles in plants include enzyme activation, stomatal control, cellular turgor maintenance, and phloem transport of photoassimilates^88^. Furthermore, it has been shown that most crops use P and K to reduce the negative effects of salt stress^5,29,31–33,39,48,68,88,89^. In the present study, the concentration of potassium (K) in shoots was similar for all salt-stressed and fertilized plants (Fig. 2B). This response was not strongly influenced by P-fertilizer types or application rates (Fig. 2B). This could be the result of supplying all treatments with equal amounts of K during the experiment. Nevertheless, the K content has not decreased in shoots of control plants while it has significantly declined in fertilized plants under salt stress conditions (Fig. 2B). Contrary to what has been reported that the decreased concentration of P and K in shoots under high salinity level is accompanied by a considerable rise in the amount of Na in shoots and roots^5,48,90^, the Na content unexpectedly increased in the shoot of fertilized plants in comparison with C+ plants (Fig. 2D). Using Ortho-A fertilizer at 30 ppm of P, the impact was more pronounced where the accumulated sodium in shoots increased by + 28% compared to C+ (Fig. 2D). The increasing sodium content in the plant media could be the cause of the rise in shoot Na^+^ concentration^91^. Correspondingly, K and Na contents (Fig. 2B,D) showed that the plant response to P shortage only (C−) was similar to their response under both stresses: salinity and P deficiency (C+), which reveals that their effects are not cumulative for these parameters. This statement was confirmed by Zribi et al.^68^ using *A. littoralis* plants. Accordingly, previous studies on the combined effects of abiotic stress indicate that the plant species and the type of stress strongly impact their interactions^68,92,93^. In this regard, it has been reported that plant growth and development are governed by the most limiting factor of growth when plants are exposed to both nutrient deficiency and salinity^94^. Interestingly, it was found that salt stress led to sodium (Na) damage, which affects root cells' ability to absorb K^5,88^. Accordingly, Behdad et al.^78^ found that K^+^ and Na^+^ may exist in competition and cause potassium shortage in the rhizosphere. The same study showed that the plasma membrane depolarization also activates the K^+^ outward rectifying channels to regulate the efflux and the influx of K^+^ and Na^+^, respectively^78^. Additionally, it was shown that sodium, at concentrations exceeding 100 mM (about 10 dS/m), significantly inhibited many enzymes involved in the vital process of plant growth^4^. In this regard, García-Ortiz et al.^95^ found that the enzymes that require potassium as a cofactor are particularly sensitive to the high sodium concentration in the medium. Our results are in line with previous reports in the literature^96^. Moreover, under salinity, potassium has been found to contribute to antioxidant metabolism regulation and the rise of the K^+^/Na^+^ ratio, minimizing the harmful effects of ROS produced during oxidative stress^5,88,89^. Thus, maintaining and increasing K^+^ levels may be a crucial factor in determining the ability of plants to tolerate salinity^11^.

As previously described, salt stress has a detrimental impact on how plants absorb and use P-fertilizers^82^. The high salt content in the soil can make it more alkaline, which can result in phosphate precipitation and reduce its availability for plant absorption^97^. However, the addition of P-fertilizers can have a positive effect on the growth and stress tolerance of plants, including those exposed to salt stress^98^. One way it does this, is by increasing the activity of superoxide dismutase (SOD) in the plant^99^. The enzyme works by converting superoxide (O_2_^−^) into hydrogen peroxide (H_2_O_2_) and oxygen (O_2_). H_2_O_2_ is less reactive than O_2_^−^ and can be further broken down by other antioxidant enzymes^100^. Some studies have suggested that polyphosphate may have a similar effect to orthophosphate on SOD activity in salt-stressed plants^101^. Recent studies also showed that polyphosphate can be accumulated in the roots of salt-stressed plants and that it may play a role in the plant's tolerance to salt stress by increasing the activity of antioxidant enzymes such as SOD^102,103^. Our findings were in line with the works conducted by Sun et al.^101^, thus the present results indicated that there is no significant variance in superoxide dismutase (SOD) activity between P treatments for Poly-B and Ortho-B at 30 ppm of P in comparison with C+ plants.

Furthermore, APX is an important antioxidant enzyme that transforms H_2_O_2_ into water and enhances ROS detoxification under stressful conditions^104^. Our results indicated that the application of specific P-fertilizers at different concentrations showed promising results in improving APX activity under salt stress conditions. Poly-B and Ortho-B fertilizers, when applied at a concentration of 30 ppm of P, exhibited remarkable increases in APX activity. Poly-B showed an increase of + 79%, indicating a substantial improvement in APX functionality, while Ortho-B performed even better with an increase of + 106% in APX activity. These findings suggest that these P fertilizers have the potential to enhance the antioxidant defense system in plants under salt stress. Additionally, the use of Ortho-A fertilizer at 45 ppm of P also yielded positive outcomes in terms of APX activity. It resulted in a significant increase of + 56.5% in APX activity compared to plants exposed to salt stress (C+). This highlights the effectiveness of Ortho-A fertilizer in improving the plant's antioxidative capacity under salt stress conditions. The results emphasize the significance of selecting the appropriate form and dosage of P fertilizers to positively influence APX activity and mitigate the detrimental effects of salt stress. These findings provide valuable insights for optimizing fertilizer strategies to enhance plant resilience and antioxidative defense mechanisms in the presence of salt stress. Moreover, the reaction of the plant to stress, including salt stress, is mediated by the protein catalase (CAT). Hydrogen peroxide, a byproduct of the stress reaction, is broken down into water and oxygen by CAT^100^. Furthermore orthophosphate (Ortho-P) has been shown to improve the activity of catalase (CAT) in plants, particularly under salt stress conditions^105^. Thus, the addition of orthophosphate to the growth medium can increase the activity of CAT, helping to protect the plant from the harmful effects of salt stress by neutralizing hydrogen peroxide^106^. Additionally, orthophosphate can help to improve the overall growth and development of plants under salt stress by increasing the uptake and utilization of other essential nutrients^107^. According to the results, the application of the P treatments to wheat plants irrigated by saline water also led to a significant increase in the CAT activity as compared with the values of control plants. Importantly, the difference in CAT activity among the different fertilizer form was significant, particularly for Poly-B fertilizer. Poly-B significantly increased CAT activity by 51% at a concentration of 30 ppm of P, when compared to the CAT activity in the salt-stressed, unfertilized plants (C+). These results demonstrate that the application of fertilizers, particularly Poly-B at a specific P concentration, can effectively enhance CAT activity and potentially mitigate the negative impact of salt stress on the antioxidative defense system of plants.

Furthermore, proline content in plants developing under salt stress has been shown to benefit from polyphosphates^108^.

In addition, our results revealed that the Ortho-B at 30 and 45 ppm, and Ortho-A at 45 ppm, enhance the proline amounts which were recovered in wheat plants grown in salt conditions and were inversely correlated with an increase in photosynthetic pigments. Moreover, the highest increase in proline content was observed when using Ortho-A at 45 ppm of P, which resulted in a + 377% increase compared to the C+ plants. Besides acting as protectors, polyphosphates can also assist in defending proline against deterioration. Reactive oxygen species (ROS), which are created when plant cells are under stress, are known to degrade proline^109^. However, polyphosphates can scavenge ROS because they could act as a cofactor for enzymes that degrade ROS, such as peroxidases, and help to regenerate glutathione, an antioxidant molecule that is important for protecting cells from oxidative damage^110^ and prevent their detrimental impacts on proline, thus the proline concentration in the plant tissue can be preserved^111^.

Moreover, soluble sugars, such as glucose, fructose, and sucrose, play important roles in plant metabolism and energy production^112^. Reactive oxygen species (ROS), which are created when plant cells are under stress, are known to degrade soluble sugars^113^. However, phosphates have the ability to scavenge ROS and stop their harmful effects on soluble carbohydrates, preserving their concentration in the plant tissue^114^.

Besides this, the advantageous effects of polyphosphates and orthophosphate, which promote root development, may lead to an increase in the uptake of water and minerals, including sugar, increasing the content of soluble sugar^115^. Therefore, our results indicate that all P treatments led to an increase in the content of soluble sugars in leaves, which is in agreement with prior studies. Furthermore, it has been noted that polyphenols, a diverse group of secondary metabolites, play significant roles in plant growth and development as well as in how plants react to different environmental stresses, such as salt stress^116^. It has been demonstrated that polyphosphates have a beneficial impact on the polyphenol level in plants undergoing salt stress^117^. By encouraging the activity of enzymes involved in the production of polyphenols, such as phenylalanine ammonia-lyase (PAL) and chalcone synthase, polyphosphates can improve the biosynthesis of polyphenols^118^. These enzymes are known to be induced in reaction to a variety of stresses, including salt stress, and are important regulators of the polyphenol biosynthetic pathway^119^. By serving as protectors, phosphates can also aid in preventing polyphenols from degrading^120^. It is well known that reactive oxygen species (ROS), which are created when plant cells are under stress, are capable of degrading polyphenols. Polyphosphates have the ability to absorb ROS and stop them from harming polyphenols, preserving their concentration in the plant tissue^121^. Moreover, our findings demonstrate that all the P treatments have a positive impact on the total polyphenols content. Specifically, at 30 ppm of P, Poly-B showed the highest significant enhancement (+ 93%) when compared to C+ plants. Likewise, polyphenols have been shown to possess antioxidant qualities and the ability to scavenge ROS, both of which can help to lessen oxidative stress and the harm that salt stress causes to plant cells^122^. This implies that the plant's overall capacity for antioxidants may be enhanced by the beneficial impact of polyphosphates on polyphenol content^123^.

In addition, one of the ways that polyphosphates can benefit plants under salt stress is by reducing the level of malondialdehyde (MDA) in the plant cells^124^. Lipid peroxidation, a process that happens when plant cells are subjected to different stresses, including salt stress. MDA is a toxic compound that can harm cell walls and hinder the growth and development of plants^125^.

Under salt stress, polyphosphates and orthophosphates can also assist in regulating ion homeostasis in plant cells^126^. Excessive salt can damage cells and cause oxidative stress by upsetting the equilibrium of ions inside and outside plant cells^125^. However, by controlling the action of ion transporters in the cell membrane, polyphosphates can aid in maintaining ion homeostasis^37^. This can help to keep cellular function and stop the accumulation of harmful ions^127^. As a result of their antioxidant properties, which help to scavenge ROS and prevent lipid peroxidation, and their capacity to control ion homeostasis, which supports cellular function, polyphosphates can reduce the amount of MDA in plants growing under salt stress^128^. Our results revealed that there is no significant variance in MDA content for all the P treatments except a significant increase in MDA accumulation for the Ortho-A at 45 ppm of P by + 72% in comparison with C+ plants. This means that, the application of P-fertilizers did not lead to a significant increase in MDA accumulation compared to the control groups (C+ and C−) under salt stress conditions. Indeed, these findings suggest that plants treated with Poly-B and Ortho-B were able to tolerate salt stress at an electrical conductivity (EC) level of 3.003 dS/m.

In summary, the polyphosphate effect is crucial for the growth and development of plants. The variety and concentration of salt, the plant species, and the soil conditions can all have an impact on how they affect plant growth under salt stress. Therefore, more studies are required to completely understand the exact mechanisms by which PolyP impact plant growth under salt stress.

## Conclusion and perspectives

The parameters related to the plant's antioxidant system and P uptake are highlighted in this study to reveal the complex relationship between salinity and P in wheat plants. Our results demonstrated that salinity caused a series of variations in the antioxidant capacity of wheat plants, at both, enzymatic and non-enzymatic levels. Remarkably, our results show a strong correlation between P uptake, biomass, and various antioxidant system parameters as well as P rates and sources. The use of soluble P fertilizers considerably enhanced the total plant performances under salt stress in comparison with C+ plants grown under salinity and P deficiency. Indeed, salt-stressed and fertilized plants exhibited a robust antioxidant system revealed by the increase in enzymatic activities of CAT and APX and a significant accumulation of TPC, Proline, and soluble sugars as well as increased biomass, CCI, leaf protein content and P uptake compared unfertilized plants. Poly-B fertilizer showed significant positive responses at 30 ppm P compared to OrthoP fertilizers at 45 ppm P which implies that PolyP fertilizers might be an alternative for the suitable management of phosphorus fertilization to improve the productivity of salt-affected soils by the PolyP quality of chelating micronutrients, releasing available phosphorus in the rhizosphere slowly and progressively which minimize both P application frequency and P losses in soil over time. Hence, our findings might help to improve the P acquisition efficiency by wheat plants and lead to better polyphosphate utilization in saline environments, where fundamental and applied knowledge is still lacking.

## Data Availability

All data generated or analysed during this study are included in this article. Excel files can be provided on demand and should be addressed to A.O. The seeds were collected from SONACOS (National seeds corporation commercialization, Morocco). The experiment has been conducted on one variety of durum wheat (cv Karim) listed in the catalogue of the same company.
